# Quantifying professionalism in peer review

**DOI:** 10.1186/s41073-020-00096-x

**Published:** 2020-07-24

**Authors:** Travis G. Gerwing, Alyssa M. Allen Gerwing, Stephanie Avery-Gomm, Chi-Yeung Choi, Jeff C. Clements, Joshua A. Rash

**Affiliations:** 1 Department of Biology, University of Victoria, Victoria, British Columbia Canada; 2 Sidney Museum and Archives, Sidney, British Columbia Canada; 3 Science and Technology Branch, Environment and Climate Change Canada, National Wildlife Research Centre, Ottawa, Ontario Canada; 4 School of Environmental Science and Engineering, Southern University of Science and Technology, Shenzhen, China; 5 Department of Biology, Norwegian University of Science and Technology, Trondheim, Norway; 6 Department of Psychology, Memorial University of Newfoundland, St. John’s, Newfoundland Canada

**Keywords:** Biology, Peer review, Psychology

## Abstract

**Background:**

The process of peer-review in academia has attracted criticism surrounding issues of bias, fairness, and professionalism; however, frequency of occurrence of such comments is unknown.

**Methods:**

We evaluated 1491 sets of reviewer comments from the fields of “Ecology and Evolution” and “Behavioural Medicine,” of which 920 were retrieved from the online review repository Publons and 571 were obtained from six early career investigators. Comment sets were coded for the occurrence of “unprofessional comments” and “incomplete, inaccurate or unsubstantiated critiques” using an a-prior rubric based on our published research. Results are presented as absolute numbers and percentages.

**Results:**

Overall, 12% (179) of comment sets included at least one unprofessional comment towards the author or their work, and 41% (611) contained incomplete, inaccurate of unsubstantiated critiques (IIUC).

**Conclusions:**

The large number of unprofessional comments, and IIUCs observed could heighten psychological distress among investigators, particularly those at an early stage in their career. We suggest that development and adherence to a universally agreed upon reviewer code of conduct is necessary to improve the quality and professional experience of peer review.

## Background

Peer review, the foundation of modern science, is the gatekeeper of scientific advancement. Theoretically, peer reviewers engage in a collegial but thorough review of a manuscript, where ideas, methods, and interpretations are constructively criticized. The goals of peer review are to ensure the credibility and integrity of the scientific record by pointing out weaknesses, offering feedback for improvement, and ensuring that misleading science is not published. Unfortunately, peer review has attracted criticism surrounding issues of efficiency, bias, and fairness [1–3]. While championed by many scientists [4], there is a paucity of empirical evidence to support the effectiveness of peer review for improving manuscripts [5–7]. Making matters worse, some peer-reviewer comments lack professional comportment, with comments demeaning authors, or focusing upon author gender, sex, race, or country of origin, rather than the technical merit of the submitted work [3, 7–9]. Unprofessional comments may contribute to psychological distress within academia, particularly among early career investigators (ECIs) [9, 10].

In a recent study, Silbiger and Stubler [3] surveyed the lifetime prevalence of unprofessional (demeaning) comments made during the peer-review process. A self-selecting sample of 1106 academic authors were recruited via social media platforms, direct posting on scientific list-serves and email invitations to colleagues, department chairs and organizations focused on diversity and inclusions in Science Technology Engineering Mathematics (STEM) fields. Over half (58%) of surveyed authors reported receiving at least one unprofessional review over their career, highlighting that academic peer review can be a deflating experience at times.

While claims of unprofessional peer-review are rampant [9], there has been a lack of quantitative assessments of reviewer comments. Data reported by Silbiger and Stubler [3] were reliant upon self-report, a method that is susceptible to sampling and response biases, and quantitative approaches are needed to expand on those observations. To fill this critical gap, we retrieved peer-reviews of academic manuscripts published in the fields of “Ecology and Evolution” and “Behavioural Medicine” and coded comments for two general themes: 1) professional comportment; and 2) incomplete, inaccurate or unsubstantiated critiques (IIUC).

## Methods

We evaluated 1491 sets of comments obtained from peer reviewers and calculated the portion that contained unprofessional comments or IIUCs. A comment set was defined as all comments provided by a single reviewer (e.g., Reviewer#1) during a single round of review. For instance, three reviewers providing feedback in one round of revision would produce three comment sets. If three more reviewers provided another round of comments, this would equal six comment sets in total. Reviewer comments obtained from subsequent rounds of revisions were treated as separate interactions with the authors, given that there was no way to guarantee that reviews were completed by the original reviewers. Peer-review comment sets were obtained from two independent sources: 1) author case studies; and 2) Publons.

### Author case studies

Three hundred twenty-seven early career investigators (i.e., having obtained their terminal degree, PhD or MSc, within 10 years) who had published in the fields of “Ecology and Evolution” or “Behavioural Medicine” within the past year were provided with information about this research through informal collegial discussion. Fifteen researchers showed interest in the project, and of those six (four from Ecology and Evolution and two from Behavioural Medicine) were able to provide reviewer comments for their published works. Invited publications were omitted. Case studies include comment sets for papers that were rejected from one or more journals but were eventually accepted for publication.

### Publons

Searches for reviewer comments were carried out on November 26th, 2019 using the open access repository of peer-review comments (Publons). Searches were constrained between 2000 and 2019, and to pre-publication comments for published manuscripts in the subjects of “Ecology and Evolution” and “Behavioural Medicine.” These subject areas were chosen to match those provided by author case studies. Ecology and Evolution papers were accessed in the Agricultural and Biological Sciences category (455,004 manuscripts available), and Behavioural Medicine in the Medicine category (4,699,984 manuscripts available). Three hundred manuscripts from each subject were randomly selected for evaluation using a random number generator (random numbers generated in Microsoft Excel [formula: randbetween1,X; where X is the number of manuscripts available from that year]). Identified manuscripts were sorted by year, and we aimed to evaluate an equal number of manuscripts, 15, from each of the 10 years between 2000 and 2019. Not all uploaded reviews had reviewer comments, therefore, the total number of evaluated manuscripts in each subject matter was lower than 300 (Ecology and Evolution: 290 manuscripts and 666 reviewer comment sets; Behavioural Medicine: 278 manuscripts, and 825 reviewer comment sets). If 15 manuscripts with comment sets were not available from a given year, the deficit was added to the subsequent year’s total. Reviewer comment sets, not a manuscript, were the base unit of replication for this study. This resulted in the selection of 568 manuscripts and 1491 reviewer comment sets.

### Blinding of assessment

Comment sets were blinded from coders (i.e., manuscript title, year of publication, journal, and author names were removed) in order to minimize the potential for bias. This prevented temporal analyses, analyses by impact factor and analyses by author gender, sex, or race, but preserved reviewer and author anonymity. We deemed this of high importance given that most reviews were conducted under the expectation of anonymity.

### Evaluation questions

Assessments were conducted by TGG and JAR using framework analysis [11]. A coding rubric was developed a-priori based on our previous work [8], and will be made available through reasonable requests made to the corresponding author. The rubric was pilot tested on 5% of reviewer comment sets to ensure agreement between coders. Inter-rater agreement was substantial following pilot testing (95% agreement of binary responses for individual questions), and each of the remaining reviewer comment sets were coded by only one author. Reviewer comments were coded across 7 domains: Response to a domain was binary. If a comment set contained an instance of a domain, it was marked as positive. Percent of reviewer comment sets positive for a domain were then presented as an overall total, as well as by subject, case studies, and from Publons comments.

#### Unprofessional comments

##### Unprofessional comments

This question evaluated how many sets of reviewer comments included unprofessional comments about the author or the work. Comments that focused on an author’s sex, gender, age, race, place of origin, or native language, as well as comments that could be interpreted as insulting or demeaning were included. Examples included statements such as: “the writing of this paper was atrocious,” “this young lady is lucky to have been mentored by the leading men in the field”, “the authors provide us with a nice example what they can, and cannot do, and how they (wrongly) understand nature and ecology,” and “the authors are clearly new to this field and it shows in this work.”

##### Questionable research practices

Some comments accused the authors of employing questionable research practices, and while we quantified this number, we cannot assess the accuracy of these claims. Examples include accusing the authors of purposely misrepresenting a study (“it is convenient that you didn’t mention …” ), collecting data using unethical methods (“methods used violate basic precepts of animal welfare”), omitting literature to create a strawman argument (“I find it impossible to believe they were unaware of the work of ….” ), or misrepresenting the data of their own study (“it leaves me with the feeling that authors present a very partial view on the topic or are just not familiar with the literature”).

#### Incomplete, inaccurate or unsubstantiated critiques

##### Inaccurate statement about clearly stated information in draft (case studies only)

Often reviewers state that authors have omitted a key piece of information in the manuscript when that information is already clearly stated. In order to limit the influence of confusing writing confounding this issue, only extreme examples were used. For instance, reviewers stating that sample size was not provided, when in fact it was clearly stated (“therefore, total sample size was X”). Examples were identified by author responses to reviews, and by assessing submitted manuscripts when one was available. This was only evaluated in case studies given that not all Publons reviews included author responses.

##### Arguments from authority

This question quantified the number of comment sets that included arguments from authority. Specifically, when a reviewer makes a claim but does not support this claim with citations or sufficient empirical or descriptive text to evaluate the claim. Examples include stating that a method is wrong without justifying that claim (“analysis was bad and made me forget what I already knew about ANOVA”), claiming authors’ interpretation of the data is incorrect without explaining why (“this is JUST wrong”), or providing an alternative interpretation of data without supporting that claim (“much more likely that predation was the driving factor”). We did not assess if the reviewer’s point was valid, instead this question evaluated whether sufficient detail was provided for the reviewer’s claim to be evaluated. This category did not include statements regarding missing literature.

##### Unaware of, or criticizing common techniques

Comments from reviewers that attacked commonly used methods that are supported by the preponderance of evidence (e.g., 5 supporting citations in previous 10 years). Comments identified as contradicting well supported methods did not include instances where reviewers asked for nuanced justification of methods (e.g. I am wondering why “x” was used instead of “y”), nor pointing out or asking for clarification about known flaws with the method. Instead, we counted instances where reviewers viewed common methods as critical flaws.

##### “Literature missing” but no citations provided

Comment sets that state that key literature is missing, but then do not provide any citations for that literature. For instance, indicating that a critical reference or comparisons to previous studies was missing without providing any information regarding missing literature. By not providing citations/details, the reviewer’s claim that important literature was missed can not be assessed.

##### Review was superficial

These reviews were superficial, and this category is the only one that evaluates the review overall, and not a specific comment (review length was not explicitly quantified). For instance, the review does not detail critical flaws; the reviewer only comments on editorial issues offering neither positive or negative comments on the validity of the work; technical issues are mentioned but not detailed; major revisions suggested with no guidance offered; more analyses are requested without detailing what those are or what is deficient in provided analyses, etc. Brief comment sets detailing identified issues, comment sets indicating that authors had sufficiently addressed previous reviewer comments, or comment sets that built upon previous comment sets, were not considered superficial.

### Ethics approval

The Memorial University of Newfoundland Interdisciplinary Committee on Ethics in Human Research (ICEHR) approved coding of peer-reviewer comments as secondary data analysis (ICEHR# 20210328-SC). Peer reviewer comments will not be made publicly available.

## Results

Overall, 12% (179) of comment sets included at least one unprofessional comment (Table 1), 41% (611) included at least one IIUC, and 43% (641) included at least one unprofessional comment and IIUC. Conversely, 57% (850) of reviews included neither unprofessional comments nor IIUCs.

**Table 1:** Percent of reviewer comment sets containing unprofessional comments or incomplete, inaccurate or unsubstantiated critiques (IIUC), divided by subject matter and by case studies (CS)

Topic	Survey Question	Overall	Ecology and Evolution						Behavioral Medicine			
		Total	Subject Total	CS1	CS2	CS3	CS4	Publons	Subject Total	CS5	CS6	Publons
Unprofessional Comments	Demeaned or Attacked the Author	12	18	35	18	9	15	10	7	4	6	7
	Accusation of Questionable Research Practices	2	3	2	8	2	3	4	2	2	3	2
IIUC	Superficial Review	19	20	24	18	17	21	19	18	1	13	22
	Inaccurate Statement About Clearly Stated Information in Draft	22	32	52	8	9	10	–	8	0	13	–
	Arguments from Authority	27	36	56	34	40	33	25	20	12	31	19
	Unaware of, or Criticizing Common Techniques that Are Supported by the Preponderance of Evidence	14	21	43	24	17	11	11	8	0	16	7
	Claims of Missing Literature with no Citations or Direction offered.	19	27	54	18	21	23	14	12	10	10	13
		1491RCS	666RCS	181RCS	38RCS	47RCS	80RCS	320RCS	825RCS	90RCS	135 RCS	600RCS
		568MS	290MS	29MS	10MS	17MS	34MS	201MS	278MS	26MS	50MS	202MS

*RCS* Reviewer comment set, *MS* Manuscript

Examples of unprofessional comments included (retaining original spelling and grammatical errors): “Only the meagerest of efforts was required to see the value, or lack there of, of this work,” “as is common from research from China,” and “utterly disapointed in this submission, it achieves nothing, and was a waste of funding (additional examples in Table S1)”. 2% (30) of comment sets included an accusation of questionable research practices; 19% (283) of reviews were superficial; 22% (328) of case study comment sets contained inaccurate statements about information clearly stated in the manuscript; 27% (402) of comment sets included unsupported authoritarian arguments; 19% (283) of comment sets stated that critical literature was missing but did not provide guidance on what that literature was; and 14% (209) of comment sets included attacks upon common methods supported by a preponderance of evidence. Variation was observed not only between subject matter, but also between case studies, and between case studies and Publons (Table 1).

## Discussion

From a sample of nearly 1500 reviewer comment sets from manuscripts published in “Ecology and Evolution” and “Behavioural Medicine,” we observed that approximately one in eight reviewer comment sets contained unprofessional comments. Previously, Silbiger and Stubler [3] observed that 58% of authors surveyed self-reported having received unprofessional comments in a review over their career. It appears as though a lack of professional comportment may have a large impact upon the experience of peer review. It is difficult to describe peer-review as collegial given the observed prevalence of unprofessional comments. It is also hard to imagine that such a high level of demeaning behaviour would be tolerated within a professional workplace context without corrective interaction.

The author of case study one received the most unprofessional comments, nearly double that of the next highest case study (Table 1). This elevated rate, beyond the overall average, is a product of four clusters of unprofessional comments associated with four manuscripts. Clusters of unprofessional comments highlight the role that editors could play to improve professional comportment. When unprofessional comments were observed, subsequent comment sets by that reviewer were qualitatively observed to often contain similar content. Removing these clusters of unprofessional comments would have substantially lowered the incidence of such comments in all case studies. Specifically, removing these four clusters would have reduced the unprofessional comments case study one received to the overall average (12%). Adopting policies at the level of the journal that enable editors to request reviewers to revise or remove unprofessional comments could help lower the incidence of such comments. We appreciate that some journals require editors to forward uncensored comments to authors. In such cases, comments from the editorial board indicating that particular comments do not represent the opinions of the editor or the editorial team would be a welcomed addition.

Only 2% of assessed comment sets included an accusation of questionable research practices. Care must be taken not to over-interpret this result, as only manuscripts that were eventually published were assessed. It is possible that such accusations have identified misconduct and resulted in justified rejection, and the manuscript never being published. Such papers would not have been assessed in our analysis. However, such accusations could carry far-reaching ramifications for the career of a researcher. In every instance in our dataset, the accusation of questionable research practices were a result of miscommunication or differences of opinion in research methodologies. While it is important that concerns about questionable research practices are communicated to editors, reviewers should proceed cautiously.

We employed five criteria to evaluate IIUCs in reviewer comment sets. Overall, two in five comment sets contained at least one IIUC. We observed that 19% of reviews were superficial, providing little useful guidance to the authors. These reviews failed to evaluate strengths and weaknesses, and/or provided no details regarding fatal flaws. Such reviews are unlikely to improve a manuscript, and the lack of detail makes it difficult to assess any of the reviewer’s claims. 22% of case study comment sets contained inaccurate statements about information clearly stated in the manuscript, such as admonishing an author for not including sample size when the sample size was clearly stated (proportion of inaccurate statements could not be rated for reviews published on Publons). Comments of this nature may imply that reviewers did not evaluate the manuscript in detail.

27% of comment sets included unsupported authoritarian arguments (not supporting claims with citations or sufficient detail to evaluate the claim), Common forms of arguments from authority were vague comments associated with experimental designs or statistical analyses. These comments often stated that the design or analyses were “wrong,” or “inappropriate” to answer experimental questions; without providing citations or explanation as to why the design/analysis was inappropriate. In the Ecology and Evolution reviewer comment sets, another common expression of this was to state that sampling units and/or data were not independent, without providing details as to why this was the case and the problematic nature of data dependence in the study evaluated. Such comments resulted in manuscripts being rejected for vague, and in some cases, arguably incorrect reasons. We suggest that reviewers explain their criticisms, ideally providing citations to support their position. If citations are not available to support their opinions, then sufficient detail should be provided for authors to evaluate the critique and prepare a reasoned response.

19% of comment sets stated that critical literature was missing but did not provide guidance on what that literature was. Such comments can be difficult to address by authors, and the lack of detail makes it difficult to assess the validity of reviewer concerns. Finally, 14% of comment sets included attacks upon common methods supported by a preponderance of evidence. This indicates that reviewers may often review outside of their areas of expertise, or do not evaluate provided references to familiarize themselves with methods. Comments identified as contradicting well supported methods did not include instances where reviewers asked for nuanced justification of methods (e.g. I am wondering why “x” was used instead of “y”). Instead, we counted instances where reviewers viewed common methods as strikes against the manuscript. For instance, in one Ecology and Evolution case, a reviewer strongly critiqued the use of Poisson regression to analyze over dispersed count data, an established method of analysis. This case highlights that not all reviewers will have the required expertise to evaluate all statistical analyses. More broadly, reviewers may not always be qualified to offer comments on all sections of a manuscript, a point that could be noted in reviewer comments.

Prevalence of unprofessional comments and IIUCs were observed to vary by subject area, within case studies, and between Plubons comments and case studies. Variation between case studies exemplifies that individual experiences with peer-review can vary greatly and compassion should be extended to those for whom this process is more negative. In almost all cases, incidence of low-quality reviews and abusive comments were higher in case studies than in Plubons comments. Differences between reviewer comments in Plubons and author case studies is unsurprising given that uploading reviews to Plubons is optional and likely prone to selection bias. Further, not all reviews are uploaded for a given manuscript. As such reviewer comments from Plubons are not diagnostic for a single manuscript; however, when assessed with case studies, they offer insight into the general nature of reviewer comments. While all evaluated manuscripts were eventually published, all reviews on Plubons were from the reviewer comment sets leading to publication in that journal. Case study comment sets, on the other hand, included reviews of rejected manuscripts that were eventually published elsewhere. For these reasons, differences between Plubons and case studies must be interpreted with caution. Finally, while differences were noted, caution is warranted when drawing contrast by subject area given that only two subject areas were evaluated.

Based upon our results, we suggest some solutions to improve the experience of peer review. First, reviewers should only comment on the technical merit of the submitted manuscript, never the author. We posit that it is never appropriate to comment on the gender, sex, age, or race of the author. A reviewer should also never assume that an author is, or is not, a native English speaker. Such comments can be offensive, and often incorrect. If editorial issues are identified, they can be pointed out without referring to personal characteristics of the author. Second, when issues are identified, reviewers must be specific when providing criticism, as well as provide references to support their points, and/or enough detail for authors to implement them. As scientists, it is not appropriate to make a claim without supporting it. We maintain that reviewers should be held to the same evidentiary standard as authors and must support their criticisms. Providing citations and/or detail regarding identified issues/missing literature enables editors and authors to assess the validity of the concern, prepare a measured response, or properly implement suggested changes. Third, reviewers should only review articles that they have the time and expertise to review thoroughly. When sections of a manuscript are outside the reviewer’s area of expertise, this should be identified. Our findings also underscore the importance of editors in mitigating unprofessional comments. When unprofessional comments were observed, subsequent comment sets by that reviewer often contained similar content. Editors must be vigilant and if allowed by their journal, screen such comments immediately. Finally, a variety of tools have been created to assess the quality of peer reviews, refer to Superchi, González [12] for a detailed review. Our coding structure offers one such method to evaluate reviewer behaviour.

Another potential option to improve peer review is a wholescale systemic change, with peer review adopting an alternative model. Several alternative peer-review models have been suggested, including the use of “as-is (paper is assessed on its initial merit with no suggested changes offered),” “double-blind (reviewer and author identity redacted),” and “total transparency (all reviewer comments and author responses made public)” models. Others have suggested the use of reviewer training [1, 2, 13]. Unfortunately, when alternative models have been assessed, they have not had measurable success in improving the peer-review process [14–17]. This is not surprising, and we argue that no model of peer review can succeed unless those within the system behave in a way that upholds the system’s integrity.

Beaumont [7] and Gerwing and Rash [8] contend that a peer review code of conduct is required to promote good reviewer behaviour while minimizing harmful behaviours. Gerwing and Rash [8] provide an example of what a peer review code of conduct could entail. Scientific codes of conduct already exist in some fields, such as for professional engineers or biologists. Unfortunately, such codes of conduct do not extend to peer review. While some journals offer guidelines around reviewer behaviour, this is far from the norm. Further, such guidelines lack the rigor of an accepted professional code of conduct [8]. Based on the findings of our investigation, we endorse the adoption of a peer review code of conduct. If an explicit code of conduct was available to guide reviewer behaviour, as well as to judge conduct against, editors would not be required to make judgement calls as often. If issues are detected with reviewer comments, Editors could request that reviewers provide feedback that conforms to the code of conduct. Therefore, assisting editors in what is admittedly a difficult job (to say nothing of finding reviewers in the first place). Finally, peer-reviewer training could be designed around such codes of conduct to provide a universal standard.

## Limitations

The results of this manuscript must be considered in light of several limitations, many of which can help guide future research in the area. First, we focused on reviewer comments, rather than author behaviours [12]. Second, reviewer behaviour was pooled across external and internal peer-reviewers which prevents a nuanced understanding about whether reviewer behaviour varied among those on the editorial board. Third, journal names were not extracted or evaluated in an attempt to preserve blinding and prevent expectancy effects. This precluded a nuanced understanding about whether reviewer behaviour varied by journal, impact factor, or peer-review policy (e.g., single/double/triple blind or open peer-review). Fourth, reviewer behaviour was only considered for “Ecology and Evolution” and “Behavioural Medicine.” Both are large fields and it was not possible to determine how close the scientific community was within areas of sub-specialization. Peer review is a human interaction and reviewer behaviour could vary by the size or interconnectedness of the community. Fifth, neither the authors gender, ethnicity, nor academic rank were quantified and assessed in the current manuscript which prevented a more nuanced understanding of whether reviewer behaviour varied as a result of demographic or occupational characteristics. Sixth, final recommendation (i.e., accept, minor/major revision, reject) was not available for many reviewer comment-sets which precluded a nuanced understanding of the degree to which reviewer behaviour varied by final recommendation. Seventh, the majority of reviewer comment sets were coded by one investigator which may raise concerns about reliability of codes despite substantial inter-rater agreement during pilot testing the rubric across 5% of reviewer comment sets. Eighth, there is a degree of subjectivity in quantifying what constitutes unprofessional and IIUC peer-reviewer comments. While based on criteria previously published [8], we realize that not everyone will agree with our criteria, and hope that this will serve as a useful starting point to a more meaningful conversation. Finally, results may be suspect to selection bias. Authors who agreed to participate in case studies are likely those who are most passionate about the issue of peer-review and may have been more likely to experience unprofessional reviewer comments, while reviewers who agree to make their reviews public on Publons may be more courteous in their responses than those who do not.

## Conclusions

Overall, 12% of assessed reviewer comments contained unprofessional comments, and 41% contained at least one IIUC. While there are many ways to potentially address this issue, we maintain that a peer-review code of conduct is a necessary first step. There are many reasons for addressing the issues with peer review as highlighted here and elsewhere [3, 9]; however, one critical reason may be to improve the mental health of those in academia. A recent editorial in Nature revealed that the mental health of ECIs is dismal, and getting worse [10]. Bullying and harassment were issues of particular concern to the mental well-being of ECIs, and underscore the need to urgently address unprofessional peer-reviewer behaviour in academia. Given the frequency of egregious examples of bullying and harassment identified in our assessment, we contend that unprofessional peer reviews may represent a source of ECI mental health issues As such, implementing a code of conduct, could help improve the experience of peer review for ECIs and all academics. .

## Supplementary information

**Additional file 1: Supplemental Table S1.** Examples of demeaning statements made by peer reviewers towards authors. Typographical and grammatical errors were retained from the author’s original comment. **Supplemental Figure S1.** Flow diagram depicting inclusion of reviewer comment sets.

## Data Availability

The datasets generated and analysed during the current study are not publicly available to protect the identity of peer-reviewers, but are available from the corresponding author on reasonable request.
