# Supplementary material for: Quantifying professionalism in peer review
Source: Res Integr Peer Rev. 2020 Jul 24;5:9. doi: 10.1186/s41073-020-00096-x (PMC7379804; doi:10.1186/s41073-020-00096-x)
Supplement: Supplementary file 1 — Additional file 1: Supplemental Table S1. Examples of demeaning statements made by peer reviewers towards authors. Typographical and grammatical errors were retained from the author’s original comment. Supplemental Figure S1. Flow diagram depicting inclusion of reviewer comment sets. [file 41073_2020_96_MOESM1_ESM.docx]

Supplemental Table S1: Examples of demeaning statements made by peer reviewers towards authors. Typographical and grammatical errors were retained from the author’s original comment.

Demeaning Statements

Only the meagerest of efforts was required to see the value, or lack there of, of this work

Utterly disapointed in this submission, it achieves nothing, and was a waste of funding

Either the authors didn't read my previous review or they are unable to grasp the subtlties

I have rewritten so much of this troubled paper that I should be included as an author

There inability to grasp even the basic elements of my previous comments makes me hesitatnt to offer even more comments as they will just not understand them

The authors clearly do not understand even the simplest aspect of…..

It was garbae when I read it first and its still garbae

Analysis was bad and made me forget what I already knew about ANOVA

Your rebuttal on the surface appears intellient but shows a tragic lack of depth and your personal limitations

Their refusal to make my suggested changes shows their poor understanding of science

The authors use a method that they developed and which so far only they use. I wonder why that is

This mansucirpt was not worth my time so I did not read it and recommend rejection

The issue addressed here is so obvious it is clear a lack of originatily will limit them

As english is clearly a problem I suggest other authors give this a good read

I tried to find something redeaming but failed to do so

I did not give a detailed review as the work was not worth it

The authors are either clearly incompenent or purposely misrepresent their work

Seems strange the authors would be unaware of this basic fact

Given the quality of this work I question the apparent qualifications of the authors

The authors clearly have little experience with these methods

The writing is aweful

I hope senior colleauges can help salvage this

nieve and simplistic view

If the authors want to publish knowing the results are unrealiable I guess that is there problem

It is clear the authors are not native enlish speakers

this is wishful thinking

consult any book about this topic

clearly the authors are following a template they picked up somewhere

as is common from research from China

if they were more fully informed they would go with the latest science, which is machine learning

basically follow a phantasy set up

this paper and its science will be another one of the hollow and non-achieving papers

the authors seem to think their goals are scientifically valid

I mean, how outdated is that

the authors provide us with a nice example what they can, and cannot do, and how they (wrongly) understand nature and ecology

With so many native English speaking co-authors, it should be easy to rectify this

authors feel forced to create artificial importance

It leaves me with the feeling that authors a very partial view on the topic or are just not familiar with the literature

Considering your methods, this is too pretentious

attempt to convince a naïve general audience that

basically cute jargon that does not add to level of understanding

that is totally distracting, just the author trying to be clever

the results of this experiment are difficult to believe.

the authors have created a novel and worthless metric that completely obscures

If there are any solid results from this experiment

the result of attending university in a developing country

this young lady is lucky to have been mentored by the leading men in the field

I’m not sure if the discussion could be even more basic

Pointless tests of widely accepted phenomenon? Maybe.

But the experimental design is decidedly 'old fashioned'

I stingily recommend that a native english speaker goes through the MS and sorts out some of the harder to understand parts.

Authors should ask for help of a colleague whose first language is English,

This is dismal to read,

Nothing new is added, why was this study even done?

The experiments to determine this is obvious, so their inability to conduct them is baffling

other methods are avilable to collect actual data

why they bother to even resubmit this is beyond me

maybe get a native English speaker to proofread the manuscript

this is worthless, hense I totally ignore

would be lucky to be published in a less prestigious journal let alone

A native english speaker would

No contrasts with other studies! They are either behind latest advnces or left these studies out t inflat their own impact

they have FINALLY incorperated my earlier comments

Another review on a a topic that has collect so many reviews that nothing new is ever added

This writing in the introduction is not high quality and should be reviewed by a native English speaker

Discussion was dull

This section was very dull

Discussion was boring

carefully proof-read by a native speaker.

in a methodologically grossly inadequate manner, which is further highlighted by the fact that the trial was unethical

Supplemental Figure S1: Flow diagram depicting inclusion of reviewer comment sets.

**Publons**

**Case Studies**

**(N=6 investigators)**

Evolution and Ecology
(n = 320)

Behavioral Medicine
(n = 600 )

Evolution and Ecology
(n = 346)

Behavioral Medicine
(n = 225)

Reviewer comment-sets coded in total
(n = 1491)

Reviewer comments from Publons
(n = 920)

Reviewer comments coded in Case Studies (n = 571)
(n =	)
